# The role of inflammation in Ischemic stroke: from biomarker to treatment

**DOI:** 10.3389/fimmu.2025.1608353

**Published:** 2025-09-26

**Authors:** Liangyi Xiao, Yini Huang, Lingying Wu, Shanshan Zeng, Changjiang Qiu, Xing Li, Le Xie, Dahua Wu

**Affiliations:** ^1^ Department of Acupuncture Rehabilitation, Changsha Hospital of Traditional Chinese Medicine (Changsha No.8 Hospital), Changsha, Hunan, China; ^2^ Department of Neurology, Hunan Provincial Hospital of Integrated Traditional Chinese and Western Medicine (The Affiliated Hospital of Hunan Academy of Traditional Chinese Medicine), Changsha, Hunan, China; ^3^ Graduate School, Hunan University of Chinese Medicine, Changsha, Hunan, China

**Keywords:** stroke, ischemic stroke, inflammation, biomarker, TCM

## Abstract

Stroke ranks among the most prevalent diseases globally. Ischemic stroke (IS), constituting 87% of all strokes, poses a significant threat to patients’ health. Following the onset of IS, within a few minutes, inflammation is initiated. This inflammation activates immune cells and related signaling pathways, further exacerbating the inflammatory state, eventually leading to irreversible brain injury. Therefore, regulating the inflammatory response can contribute to the treatment of IS. This review delves into the inflammatory mechanism in IS, the role of inflammatory markers, and the research advancements regarding the use of inflammatory markers in treatment. While previous studies often concentrated on a single aspect of the inflammatory mechanism or specific inflammatory markers, this review systematically synthesizes the fragmented information. It offers readers a comprehensive and coherent view, facilitating an in - depth comprehension of the complexity of the inflammatory response in IS. This article not only expounds the value of inflammatory markers in disease diagnosis and prognosis, but also highlights the research progress including traditional Chinese medicine treatment. This has important guiding significance for clinicians to formulate precise treatment plans, and provides a variety of options for clinical practice. The research progress of these treatment strategies presents new opportunities for addressing the challenges in IS treatment, with the potential to improve patients’ prognosis and quality of life. Through a comprehensive overview of inflammation - related studies in IS, this review serves as a valuable reference for research and clinical practice in this field, contributing to the further development of IS treatment research.

## 1 Introduction

Stroke primarily results from the rupture or occlusion of cerebral blood vessels, which disrupts the normal blood supply to brain tissue, thereby inducing corresponding cerebral injury and neurological deficits. It remains one of the most widespread and debilitating diseases affecting the global population (1). A report indicates that in 2016, stroke prevalence in the United States is 2.5%, affecting approximately 7 million individuals (2). IS mortality in China is 30% and 70% of survivors suffer from sequelae (3). An estimate suggests that the annual cost of stroke during 2014–2015 was $45.5 billion (4). Among various types of stroke, approximately 87% are IS, making ischemia the most common cause (5).

IS is characterized by a series of neuropathological processes, in which a robust and persistent inflammatory response is a key factor in the progression of brain injury. The inflammation following ischemia is associated with the acute impairment of the blood brain barrier (BBB), leading to poorer neurological outcomes. In the initial stage of IS, neuroinflammation aggravates brain damage, however, the inflammatory response may promote neural repair at later stages (6). IS not only trigger local inflammation in the brain but also rapidly induce a strong systemic inflammatory response syndrome(SIRS), characterized by the release of inflammatory mediators and the activation of peripheral immune cells. Emerging evidence suggests that this peripheral inflammation is not merely a consequence of central nervous system (CNS) injury but actively contributes to neuroinflammation (7). Peripheral cytokines, chemokines, and infiltrating leukocytes can exacerbate neuroinflammation by crossing the compromised BBB or signaling through alternative pathways. Recent studies have implicated dysregulation of the gut-brain axis, characterized by altered gut microbiota composition and permeability, as a significant contributor to peripheral inflammation that can modulate CNS inflammation following a stroke.

This article comprehensively details the inflammatory mechanisms in IS, underlines the significance of related inflammatory markers in the diagnosis and treatment of IS, and also emphasizes the integration of multi - domain knowledge, including traditional Chinese medicine approaches. A profound understanding of the neuroinflammatory response in IS will provide a solid foundation for the development of innovative ideas and novel intervention strategies.

## 2 Pathophysiology of inflammation in IS

Neuroinflammation is recognized as a pivotal component in the pathophysiology of IS. A systematic integration and in depth exploration of the inflammatory response mechanism in IS hold the potential to offer novel concepts and therapeutic targets for the treatment of IS.IS typically occurs when a blood vessel supplying the brain becomes occluded. The abrupt interruption of blood circulation leads to hypoxia in brain tissue, which triggers an inflammatory response. This state disrupts the ionic balance, giving rise to neuronal excitotoxicity, oxidative stress and lipid peroxidation, ultimately culminating in neuronal damage and irreversible neurological impairment (8).

### 2.1 Inflammatory cell activation

Inflammatory cells are activated after cerebral ischemic injury. Microglia, astrocytes, neutrophils, and monocytes show different characteristics of subtypes, which play pro-inflammatory and neuroprotective functions respectively. Studies have shown that different functional subtypes are related to the stage of the disease, so how to reverse the unfavorable situation is the focus of attention.

#### 2.1.1 Resident cells of the CNS

Microglia, brain-resident macrophages, maintain immune surveillance and synaptic pruning under normal conditions. Following IS, they polarize into M1 (pro-inflammatory) and M2 (anti-inflammatory) phenotypes. M1 microglia secrete proteases, cytokines, and reactive oxygen species (ROS) via p38 MAPK, Notch, and JAK2/STAT3 pathways, exacerbating BBB disruption and neuroinflammation. In contrast, M2 microglia phagocytose debris and release neurotrophic factors to promote repair (9–13). Additionally, *in vitro* and *in vivo* studies have demonstrated that gene regulation promotes M2 polarization, which may be related to the inhibition of the TLR4/NFκB/NLRP3 signaling pathway (14–16).

Recent research has introduced a novel M2 microglia-targeting lipid nanoparticle (MLNP) method, which selectively delivers mRNA encoding phenotype-switching interleukin(IL) -10to the ischemic brain. This approach facilitates the transformation of microglia into the M2 phenotype, thereby alleviating neuroinflammation and restoring the BBB compromised by ischemia (17). Additionally, TEPP-46, an agonist of the pyruvate kinase isozyme type M2 (PKM2), has been shown to promote the transition of microglia from the M1 to the M2 phenotype following ischemia/reperfusion injury by inhibiting microglial glycolysis (18). While it is understood that microglia exhibit opposing roles at various stages of cerebral infarction (CI), the phenotypic transition from M1 to M2 is likely critical for patient prognosis. However, the time-dependent nature of this transition complicates the identification of an optimal treatment window. Furthermore, the transformation of microglial phenotypes is intricately linked to the surrounding microenvironment, including interactions with various inflammatory cell types (19). These aspects represent significant challenges that future research must address.

Astrocytes maintain BBB integrity and neurovascular homeostasis. After IS, reactive astrocytes polarize into two subtypes, each associated with distinct functions that correlate with the disease stage. In the acute phase, A1 reactive astrocytes secrete pro-inflammatory cytokines such as vascular endothelial growth factor, matrix metalloproteinase, and lipid carrier protein-2, which exacerbate damage to endothelial cells and disrupt tight junctions. Conversely, A2 reactive astrocytes produce protective factors for endothelial cells, including pentacyclic triterpenoids 3, Sonic hedgehog, and angiopoietin 1. During the recovery phase of IS, both A1 and A2 astrocytes contribute to glial scar formation, albeit through different mechanisms. A1 astrocytes facilitate glial scar formation and inhibit axonal growth via glial fibrillary acidic protein, chondroitin sulfate proteoglycan, and transforming growth factor-β (TGF-β), while A2 astrocytes promote axonal growth through platelet-derived growth factor and play a critical role in vascular remodeling (20).Recent studies have identified that 3-hydroxyquinazoline (3-HKA) can inhibit the activation of A1-type (neurotoxic) astrocytes and promote the polarization of A2-type (neuroprotective) astrocytes, thereby enhancing vascular remodeling post-stroke (21). Furthermore, the latest research has revealed a strong correlation between Lcn2 levels and the polarization of astrocyte phenotypes. By inhibiting Lcn2 to modulate astrocyte phenotype, neuroinflammation can be alleviated, and the repair of the BBB can be promoted (22). Unlike microglia, the “double-edged sword” effect of astrocytes is manifested in both spatial and functional dimensions: different functions may yield both beneficial and detrimental outcomes, complicating regulatory mechanisms. Thus, it is imperative to clearly define the disease stage and employ tailored regulatory strategies for astrocytes. However, whether in experimental or clinical studies, the delineation between the acute and recovery phases remains ambiguous, posing significant challenges for future research.

#### 2.1.2 Recruitment of immune cells at the periphery

Following a stroke, there is a significant increase in the release of peripheral inflammatory markers. This phenomenon occurs not only in the damaged brain tissue but also within the peripheral circulatory system. Urinary tract infections and pneumonia are among the most common complications following a stroke and represent significant sources of peripheral inflammation (23, 24). These peripheral inflammatory responses play a crucial regulatory role in the immune inflammatory response post-stroke, promoting the development of CI and influencing both the severity and progression of the disease.

Neutrophils rapidly infiltrate ischemic brain tissue within 6–12 hours post-stroke, peaking at 2–7 days and persisting for ≥2 weeks. They exacerbate injury by releasing neutrophil extracellular traps (NETs), inflammatory mediators, and matrix metalloproteinases (MMPs), which disrupt the BBB and promote neurotoxicity (25–29).Neutrophils exhibit dual phenotypes: pro-inflammatory N1 cells secrete harmful substances, while protective N2 cells enhance clearance by macrophages and reduce infarct volume. Ischemic neurons induce N1 polarization via conditioned medium, increasing NET formation. TLR4-deficient mice show augmented N2 infiltration and reduced injury, suggesting TLR4 modulates neutrophil phenotype toward protection (30–32).

Lymphocytes play a crucial role in the development of stroke. T cells migrate to ischemic brain regions within 24 hours of stroke, with CD4+ and CD8+ subsets releasing cytotoxic proteins (granzyme/perforin) and cytokines (interferon-γ/IL) that damage neurons and disrupt BBB integrity (33–35). Regulatory T cells (Tregs), however, correlate with reduced infarct volume and improved outcomes via amphiregulin - mediated suppression of neurotoxic astrocyte proliferation. Treg deficiency increases apoptotic neurons and astrocyte neurotoxic gene expression, reversed by Treg adoptive transfer (36–38).

Monocytes migrate to ischemic brain regions early after stroke, with classical monocytes secreting pro-inflammatory cytokines [IL-1/6/tumor necrosis factor(TNF)-α] and MMP-2/9 that disrupt BBB tight junctions and exacerbate injury via CCL2/CCR2 and P2X4R pathways (39–41). Non-classical monocytes secrete IL-10 to counteract inflammation and promote repair (42). Ly6Chi monocytes differentiate into M1 (pro-inflammatory) or M2 (anti-inflammatory) macrophages: M1 produces IL-6/IL-1β/TNF-α, while M2 releases angiogenic cytokines. This M1→M2 phenotypic switch occurs by day 7 post-stroke, suggesting therapeutic potential in accelerating this transition to improve outcomes (43–48).

Neutrophils, lymphocytes, and monocytes are not merely isolated inflammatory cells; rather, they are pivotal in elucidating the pathophysiology of CI and in the development of novel therapeutic strategies. These peripheral recruited immune cells are advantageous due to their accessibility for sample collection, which significantly benefits clinical research. Recent clinical observations indicate that the Neutrophil-to-Lymphocyte Ratio (NLR) and the Lymphocyte-to-Monocyte Ratio (LMR) independently influence the poor prognosis of elderly patients with Acute Ischemic Stroke (AIS). Notably, the area under the ROC curve (AUC) for the combination of NLR and LMR surpasses that of NLR and LMR alone, suggesting that integrating these two indicators enhances predictive accuracy for clinical outcomes in elderly AIS patients (49). Furthermore, the relationship between these inflammatory cells and the brain-gut axis represents an important avenue for contemporary research on CI. Recent studies have shown that gut microbiota significantly influences neutrophil activation in middle cerebral artery occlusion(MCAO) mice, thereby affecting stroke severity. This research implies that targeting the microbiota with antibiotics may mitigate the exacerbation of disease linked to activated neutrophils in stroke patients; however, this hypothesis requires validation through clinical trials (50). Additionally, integrating these peripheral immune cells with factors such as age, gender, underlying diseases, and comorbidities in patients with CI can yield further insights into their interconnections. Moreover, dynamically observing the changes in these inflammatory markers throughout the progression of CI is essential for a comprehensive understanding of the disease.

### 2.2 Exosomal vesicles

EVs are lipid bilayer nanoparticles secreted by cells, playing a crucial role in mediating neuro-immune communication following ischemic events. EVs released by microglia, astrocytes, and infiltrating immune cells carry proteins, miRNAs, and inflammatory factors, and they bidirectionally regulate injury and repair processes. Vesicles derived from choroid plexus epithelial (CPE) cells contain miR-155-5p, which inhibits the expression of Ras homolog enriched in brain (Rheb) and promotes NLRP3-mediated inflammasome activation, thereby exacerbating cerebral ischemic injury (51). Conversely, numerous studies have demonstrated that in rodent IS models, EVs treatment inhibits the polarization of macrophages and microglia towards pro-inflammatory (M1) phenotypes or promotes anti-inflammatory (M2) phenotypes, thereby improving neurological outcomes (52, 53). Research indicates that M2 microglia deliver miR-124 and TGF-β, which inhibit inflammatory responses and promote neuronal survival (54, 55) Additionally, EVs derived from mesenchymal stem cells (MSCs) and adipose-derived stem cells (ADSCs) alleviate neuroinflammation and inhibit cell apoptosis and pyroptosis through the TLR4/NF-κB pathway and NLRP3/GSDMD signaling pathway (56, 57). However, some studies have reported that in rat ischemia-reperfusion models, MSC-derived EV treatment does not significantly affect the infiltration of immune cells (including CD45^+^ white blood cells, neutrophils, dendritic cells, macrophages, monocytes, and lymphocytes) in the ipsilateral parenchyma (58, 59). In conclusion, while the extracellular vesicle delivery system may represent a potential therapeutic strategy for IS, controversies persist. The unclear routine usage methods, diverse transplantation doses, and varying observation time points pose significant challenges to its widespread clinical application.

### 2.3 Activation of inflammasome

**Figure 1:**
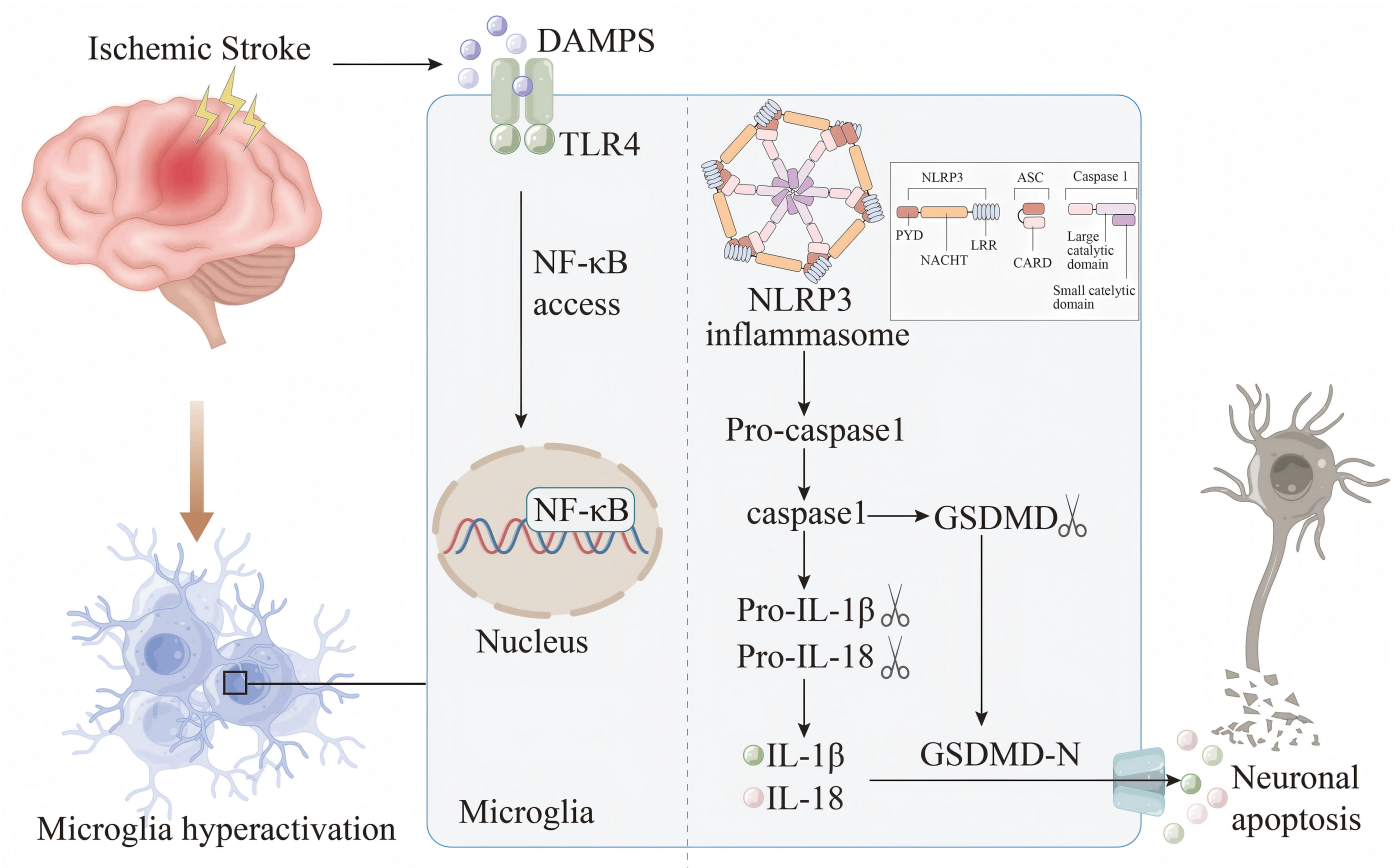
Damp-associated molecular patterns (DAMPs) are released by damaged or necrotic brain cells after IS. DAMPs are subsequently recognized by the TLR4 receptor and trigger the NF-κB pathway.This activation facilitates the assembly and subsequent activation of the NLRP3 inflammasome, setting off a cascade that results in the maturation and secretion of IL-1β and IL-18. Meanwhile, the activated caspase-1 cleaves gasdermin D (GSDMD) to produce GSDMD-N. The active form GSDMD-N is then transported to the plasma membrane, where it interacts with intimal lipids to create pores in the plasma membrane. This process leads to the release of intracellular inflammatory cytokines, which exacerbates the inflammatory response, disrupts the BBB, induces cell apoptosis, and contributes to the progression of IS.

Inflammasomes are a complex of diverse proteins that play a crucial role in glia - mediated inflammation within the nervous system. These complexes are involved in the assembly of cytoplasmic pattern - recognition receptors and are essential components of the innate immune system. The function of these inflammasomes is to recruit and activate pro - inflammatory caspase - 1, thereby influencing the maturation and release of cytokines and other inflammatory mediators, including IL-1β and IL-18 (60–62). A critical step in the formation and activation of the NLRP3 inflammasome involves the TLR4/NF - κB signaling pathway (**Figure 1**). Studies have shown that multicompound can inhibit TLR4/NF - κB signaling pathway, thereby preventing the activation of NLRP3 inflammasome in microglia, and then down-regulating the expression of downstream ACS and caspase-1, reducing the release of pro-inflammatory factors IL-1β, IL-18 and TNF-α, and ultimately alleviating ischemic brain damage (63, 64). In recent years, both *in vivo* and *in vitro* experiments have demonstrated that directly targeting NLRP3 can alleviate neuroinflammation associated with stroke. The pathways involved include SIRT3-NLRP3, FPR1/NLRP3, and the NLRP3/caspase-1/IL-1β signaling axis (65–68). In summary, the activation of the inflammasome and the release of pro-inflammatory cytokines are critical factors contributing to further damage during the pathological process of IS, mediated by microglia. Investigating the upstream and downstream signals of inflammasome activation and their interaction mechanisms will aid in the development of new regulators, addressing the limitations of currently available IS therapies and offering new hope to patients.

### 2.4 Brain-gut axis

In recent years, numerous studies have demonstrated that following brain injury, brain-derived damage-associated molecular patterns (DAMPs) enter the systemic circulation through the compromised BBB. Immune cells recognize these DAMPs via pattern recognition receptors, such as Toll-like receptors (TLRs), thereby activating both systemic and local inflammatory responses in the intestine (1). Dysbiosis of the gut microbiota contributes to neuroinflammation and peripheral immune responses post-stroke. Alterations in the microbiota-gut-brain axis (MGBA) subsequent to stroke can ultimately disrupt the gut microbiota and increase intestinal permeability. Such changes may facilitate the translocation of bacteria from the gut to sterile organs, further exacerbating the incidence of stroke-related infections (69, 70). Research indicates that gut microbiota significantly influence neutrophil activation after stroke, directly correlating with stroke severity (50). Additionally, disruption of the gut microbiota impacts the differentiation of intestinal dendritic cells and alters the balance between gut Treg cells and γδ T cells. This imbalance results in a reduction of Treg cells that produce the anti-inflammatory cytokine IL-10 in the gut, alongside an increase in γδ T cells that migrate to the brain injury site and release pro-inflammatory factors such as IFN-γ and IL-17 (71). Furthermore, vagus nerve dysfunction impairs communication between the gut and the brain, exacerbating metabolic imbalances, neuroinflammation, and cognitive impairments following stroke (72). In summary, the brain-gut axis offers a novel perspective for understanding the pathophysiological mechanisms underlying CI, highlighting the significant impact of the gut microbiota on brain outcomes. Although much of the current research in this domain remains at the basic research stage, its therapeutic potential is substantial. Regulating the gut microbiota to enhance outcomes in CI represents a promising treatment strategy.

## 3 Inflammatory markers

Inflammatory markers are detectable substances generated by cells or present in plasma during an inflammatory reaction. These markers reflect the presence, extent, and nature of inflammation within the body and are of great significance for the diagnosis, monitoring, and prognostic evaluation of inflammatory diseases. A further exploration of the role of inflammatory markers in IS may offer novel insights for the treatment of IS.

### 3.1 Cytokines

A diverse range of cytokines plays crucial roles in IS, exhibiting both pro-inflammatory and anti-inflammatory properties. Pro-inflammatory cytokines, such as TNF-α, IL-1, and IL-18, contribute to infarct expansion and neuroinflammation, while anti-inflammatory cytokines, including IL-10, IL-2, IL-4, and TGF-β, exert neuroprotective effects (73). Notably, some cytokines, such as IL-6, exhibit context-dependent roles across different phases of IS. The ideal treatment strategy is to precisely inhibit the pro-inflammatory trans-signaling pathways while retaining or even enhancing their beneficial classical signaling pathways. Although the use of soluble gp130 (sgp130) to selectively inhibit trans-signaling pathways is a promising direction (74), achieving this precise regulation *in vivo* remains challenging. Recently, employing the B/T ratio to determine the time window value for IL-6 signaling in regulating inflammation has attracted significant attention (75). In addition to the challenges associated with intervention during the time window, individual differences also influence the role of IL-6 in neurological functional outcomes. Therefore, future IL-6-targeted therapies are unlikely to adopt a “one-size-fits-all” approach; individualized treatment strategies must be considered (**Tables 1**, **2**).

**Table 1:** The role of cytokines in IS.

Main effection	Cytokines	Mechanism	Correlational research	Method	Conclusions	References
Pro-inflammatory effect	1L-1	Induces the production of toxic mediators such as TNF-a, IL-6 and cyclooxygenase-2 (Cox-2)	IL-1β plays a crucial role in facilitating NF-kB activity and the transcription of COX-2 in BBB cells in reaction to both systemic and localized inflammation, although this is not the case during endotoxemia.	animal experiment	Disruption of the BBB; Increased cerebral edema and cerebral infarction volume	(76)
		p38 mitogen-activated protein kinase (MAPK) signaling pathway in hippocampal neurons; Activation of NF-κb signaling pathway in astrocytes	Cell type-specific IL-1β signaling in the CNS.	*In vivo* experiment	Aggravates inflammatory response and brain damage	(77)
		neutrephil infiltration	A systemic inflammatory trigger enhances the acute phase along with CXC chemokine reactions to experimental stroke and intensifies brain injury through mechanisms dependent on IL-1 and neutrophils.	animal experiment		(78)
	IL-27	Induce inflammatory cytokines and promote the infiltration of inflammatory cells (in the acute phase)	Interleukin-27 deletion has neuroprotective effects in the acute ischemic stage of cerebral infarction.	*In vivo* experiment;animal experiment		(79)
	1L-18	The expression of matrix metalloproteinase (MMP) was induced	The production of IL-18 and the presence of functional IL-18 receptors in human vascular endothelial cells, smooth muscle cells, and macrophages: significance for the development of atherosclerosis.	*In vivo* experiment	Leads to atherosclerotic rupture and promotes thrombosis	(80)
	TNF-α	Involved in endothelial cell necrotic apoptosis induced by M1-type microglia	Microglia-derived TNF-α mediates endothelial necroptosis aggravating BBB disruption after cerebral ischemia.	animal experiment	Aggravates the necrosis of endothelial cells and destroyed the BBB	(81)
anti-inflammatory action	IL-33	Activate IL-33/ST2 Signaling in monocyte-derived macrophages	IL-33/ST2 signaling in monocyte-derived macrophages maintains blood-brain barrier integrity and restricts infarctions early after ischemic stroke	animal experiment; *In vivo* experiment	maintains blood-brain barrier integrity ;restricts infarctions early after ischemic stroke	(82)
	IL-10	Activation of PI3K and STAT3 decreased IFN-g, IL-1β and TNF-a	IL-10 directly protects cortical neurons by activating PI-3 Kinase and STAT-3 Pathways	*In vivo* experiment	Anti-inflammatory effect,reduces the volume of IS and participates in immune regulation	(83)
		Reduces the number of T cells and monocytes in the brain parenchyma	IL-10 producing B-Cells limit CNS inflammation and infarct volume in experimental stroke.	animal experiment		(84)
	IL-2	Increase the number of TreGs and promote the expression of CD39 and CD73	IL-2/IL-2 antibody complexes facilitate the *in vivo* proliferation of regulatory T cells, offering protection from transient cerebral ischemia.	animal experiment	Reduces the volume of IS, inhibit neuroinflammation, and promote the recovery of neurological function	(85)
		Both intracranial CD8+ T cell infiltration and peripheral CD8+ T cell activation were reduced	IL-2mab reduces demyelination after focal cerebral ischemia by suppressing CD8(+) T Cells	animal experiment	Reduces the volume of IS, reduce demyelination and improve nerve function	(86)
	IL-4	Induced microglia/macrophage M2 phenotype	IL-4 Is essential for Microglia/Macrophage M2 polarization and Long-Term recovery after cerebral ischemia.	animal experiment	Improves nerve function	(87)
		Secrete exosomes containing miRNA-26a	IL-4-Polarized BV2 microglia cells promote angiogenesis by secreting exosomes	*In vivo* experiment	Promotes angiogenesis, Reduce brain damage	(88)
	TGF-β	The toxicity induced by nmda was decreased.	GDNF and neublastin protect against NMDA-induced excitotoxicity in hippocampal slice cultures	*In vivo* experiment	Anti-inflammatory, protective neurons	(89)
			Neuroprotection facilitated by glial cell line-derived neurotrophic factor: participation of diminished NMDA-evoked calcium entry through the mitogen-activated protein kinase signaling pathway.			(90)
		Inhibit the accumulation of lipid droplets	TGF-β1 decreases Microglia-Mediated Neuroinflammation and lipid droplet accumulation in an *In vitro* stroke model			(91)
		Promote M2 polarization of microglia	The complexes of IL-2 and IL-2 antibodies promote the growth of regulatory T cells *in vivo*, providing defense against transient CI.			(55)

**Table 2:** The dual role of IL-6 in IS.

Dual-action cytokines	Main effection	Disease staging	Core effect	Cell source	Receptor subtype	Mechanism	Reference
IL-6	Pro-inflammatory effect	Several hours to several days (acute phase)	Aggravating inflammatory damage, disrupting the blood-brain barrier, and being associated with a poor prognosis	Monocytes, neutrophils, microglia, infiltrating macrophages	Soluble IL-6R	Activate the JAK/STAT3 signaling pathway	(74)
	anti-inflammatory action	Several days to several weeks (subacute phase/recovery phase)	Proliferation of neural progenitor cells, differentiation of neurons, angiogenesis and functional recovery	Neurons, astrocytes	Soluble IL-6R	Promote STAT3 phosphorylation and the transcription of pro-regeneration genes mediated by it	(92)
				astrocyte	Soluble IL-6R	Increase the expression of IL-10, TGF-β, and IL-4; reduce the expression of IFN-γ	(93)
				monocyte	Membrane-bound IL-6Rα	Induce microglia to transform into RAMs and express VEGFA	(94)

### 3.2 Chemokines

**Figure 2:**
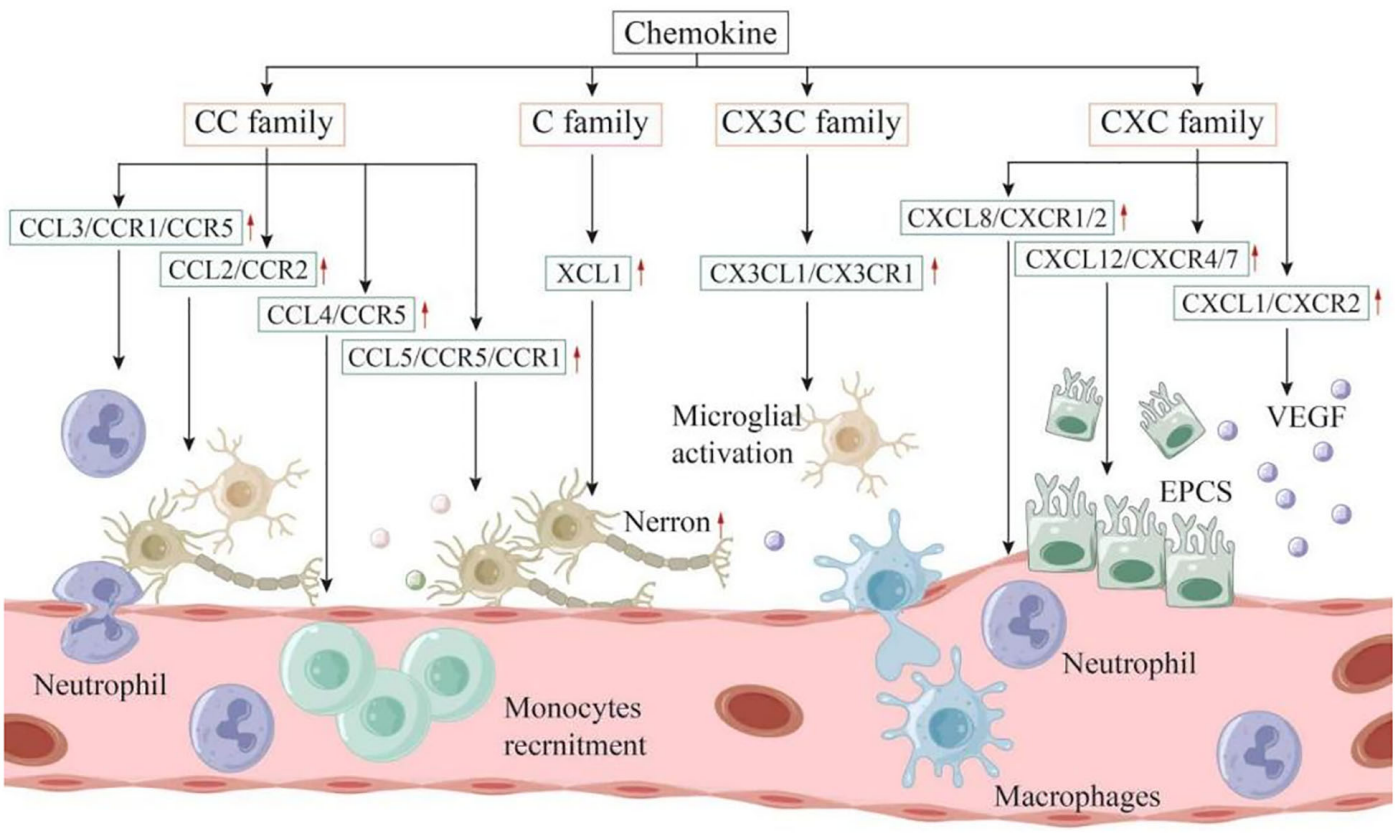
Chemokines can be categorized into four families: CXC, CC, CX3C, and C. These molecules play a dual role in both the onset and progression of IS. Within the inflammatory linked to IS, it is primarily the microglia located in the brain, along with infiltrating immune cells, that produce chemokines. These chemokines contribute to the further recruitment and activation of leukocytes, exacerbating the inflammatory response. On the other hand, following the acute phase, chemokines may also recruit neural progenitor cells, endothelial progenitor cells, and other types of cells that facilitate nerve repair and angiogenesis, ultimately enhancing the neurologic function of the patient.

Chemokines were initially identified as factors that attract circulating leukocytes to sites of inflammation or injury (95). The role of chemokines in IS is two - fold, presenting both advantages and disadvantages. Chemokines can be categorized into four families, namely CXC, CC, CX3C, and C, based on the number of amino acids separating their first two cysteine residues (96). Nevertheless, research on C - family chemokines is still limited. To date, only one review has suggested that XCL1 is involved in the interaction of neuroimmune cells and can increase the number of neurons, indicating a potential association among XCL1, neurogenesis, and angiogenesis (97). Subsequently, the relationships between the CC, CXC, and CX3C families and IS are summarized as follows (**Figure 2**).

#### 3.2.1 CC family

CCL2/Monocyte chemotactic protein-1(MCP-1) is upregulated in ischemic penumbra after injury, promoting neuroinflammation by activating microglia and mediating BBB disruption via downregulating tight junction proteins (e.g., occludin, ZO-1). While CCL2/CCR2 antagonists reduce M1 polarization and neuronal damage, CCL2 may also facilitate oligodendrocyte generation and stem cell recruitment, suggesting dual roles in injury and repair (98–101). CCL3 (MIP-1α) drives neutrophil recruitment via CCR1/CCR5, correlating with TNF-α and insulin levels in human studies (102–107). CCL4 (MIP-β) binds CCR5 to recruit monocytes, with elevated serum levels predicting stroke risk (108, 109). CCL5 (RANTES) exhibits paradoxical effects: CCL5 deficiency reduces infarct volume and BBB permeability, yet CCR5 knockout mice show worse injury outcomes, implying protective roles via neuronal survival pathways (110–114). These findings highlight complex, context-dependent functions of CC chemokines in IS.

#### 3.2.2 CXCL family

CXCL1, also known as growth - regulated oncogene (GRO) - α, acts as a chemotactic factor for neutrophils. An animal study investigated that mice with the CXCL1 - infused sponge exhibited a significant reduction in neuroinflammation and infarct size, lower mortality rates, and better neurological function following thromboembolic (TE) compared to those with a sponge infused with 0.9% NaCl (115). An *in vitro* study showed that microglia inhibited CXCL1 expression in astrocytes through IL-1RA secretion; In addition, animal studies suggest that anti-CXCL1 neutralizing antibody treatment or administration of recombinant IL-1RA protein can reduce the volume of CI and improve the motor coordination ability of mice after ischemia (116).Additionally, anti-atherosclerotic therapies, including candesartan and pioglitazone, were observed to decrease CXCL1 mRNA levels during CI (117). Moreover, activation of the CXCL1/CXCR2 signaling pathway in conjunction with thrombin and thrombin receptor agonist peptides has been shown to protect astrocytes against ceramide - induced apoptosis (118).

CXCL8 (IL-8) promotes neutrophil infiltration and neuroinflammation after CI, exacerbating ischemic injury via NF-κB activation. IL-8 knockout mice exhibit reduced inflammation through PI3K/Akt signaling. Clinically, serum IL-8 correlates with early disability in AIS, suggesting its potential as a severity marker (119–124).

CXCL12/CXCR4 signaling drives recruitment of neural/endothelial progenitors to ischemic sites, enhancing angiogenesis and neurogenesis. Drug-induced CXCL12 upregulation improves recovery in MCAO models by promoting stem cell migration and growth factor expression. CXCL12 also supports neural precursor survival via CXCR7, with plasma levels predicting future stroke risk (125–128).

Clinical studies have shown elevated serum CXCL16 levels are closely associated with atherosclerotic IS (129).Moreover, another study demonstrated that elevated serum CXCL16 levels in individuals with carotid artery stenosis after AIS were significantly associated with microembolic signals, suggesting that high CXCL16 concentrations could serve as a biomarker for predicting stroke occurrence and may be involved in plaque instability (130).

CXCL10 recruits T cells/NK cells and mediates 67% of IL-6 signaling effects on atherosclerotic stroke risk, linking genetic IL-6 downregulation to reduced CXCL10 and lower cardiovascular disease risk (131). CXCL2 upregulated in ET-1 stroke models at 24-48h post-ischemia. Microglial lncRNA U90926 knockdown reduces neutrophil infiltration via CXCL2 downregulation, highlighting its role in ischemic inflammation (132, 133).

*In vitro* studies showed that anti-CXCL5 antibody promoted the proliferation and decreased the permeability of oxygen-glucose deprivation (OGD) model cells, as indicated by the improvement of transepial electrical resistance (TEER), suggesting that silencing CXCL5 attenuates ischemia/hypoxia-induced damage to human brain microvascular endothelial cells (BMECs).Moreover, the p38 inhibitor SB203580 significantly reduced the activity of CXCL5 in the model cells, suggesting that CXCL5 may influence BMECs function via the p38 pathway (134).

In summary, the CXCL chemokine family, including several key molecules such as CXCL1, CXCL8, CXCL12, and CXCL16, plays a significant role in the onset and progression of CI and is strongly associated with atherosclerosis.

#### 3.2.3 CX3C family

CX3CL1, also known as Fractalkine, is the sole member of the CX3C chemokine family and functions as a transmembrane chemokine. This protein is synthesized by neurons and is crucial for communication between neurons and glial cells. Notably, CX3CL1 is unique within its family in that it interacts exclusively with a single receptor, CX3CR1 (135). During IS, CX3CR1 influences several factors, including the volume of the infarct area, the integrity of the BBB, angiogenesis, and neural recovery. A study showed that CX3CR1 knockout (KO) mice exhibited a larger damage area compared to both wild - type (WT) and CCR2 KO mice 48 hours post - stroke. In the chronic phase, these same CX3CR1 KO mice displayed reduced brain injury/BBB degradation and enhanced angiogenesis, thereby facilitating recovery (136). The loss of CX3CR1 reduces the recruitment of monocytes, the proliferation of microglia, and the inflammatory capacity of these two cell types (137).

### 3.3 Other inflammatory mediators

#### 3.3.1 hs - CRP

High-sensitivity C-reactive protein (hs-CRP) has been extensively studied in acute infection or acute phase of disease, but lacks specificity (138). Studies have shown that the level of hs-CRP is related to the volume of CI (139, 140) and disease outcome (141), and people with high levels of hs-CRP may be at risk of IS (142).

#### 3.3.2 MMPs

MMPs constitute an important group of proteolytic enzymes capable of degrading all components of the extracellular matrix (143). MMPs can damage the BBB and promote nerve recovery in the acute phase and recovery phase of IS, respectively (144). In the acute stage of IS, MMPs impair the integrity of the BBB by degrading the basement membrane and compromising blood vessel stability. Palm et al. (145) found that there was a correlation between serum MMP-8 concentration and the severity and etiology of stroke in patients with AIS. Twenty - one days post - stroke, an increase in MMP - 2 expression was observed in astrocytes located at the periphery of the peri - infarction region, where the ends of these astrocytes were in contact with blood vessels, suggesting a potential role for MMP - 2 in the repair processes following inflammatory injury (146). MMP-9 expression peaks within 24 hours post-IS in rats, exacerbating BBB disruption and hemorrhagic transformation when combined with tPA (147, 148). Other MMPs (MMP-2, MMP-3, MMP-10, MMP-13) also increase post-stroke, with MMP-3 linked to improved neurological recovery. MMP inhibition reduces infarct size and edema, while MMP-9 knockout mice show smaller infarcts compared to wild-type controls. MMP-2 may facilitate neovascularization, as MMP-2 knockout does not affect infarct size. Serum MMP-9 levels correlate with initial stroke severity and clinical outcomes, suggesting its potential as a prognostic marker (149–155).

#### 3.3.3 S - 100β

S-100β, a calcium-binding protein secreted by astrocytes, promotes neuroinflammation after brain injury by stimulating astrocyte/microglial release of inflammatory factors and nitric oxide. Elevated cerebrospinal fluid/serum S-100β correlates with AIS severity and neurological impairment. Animal studies demonstrate that reducing S-100β levels via pharmacological or genetic interventions decreases infarct volume and improves functional outcomes in MCAO models. These findings highlight S-100β as a potential therapeutic target and prognostic biomarker for IS (156–166).

#### 3.3.4 NSE

Neuron - specific enolase (NSE) is a glycolytic enzyme present in cells. Specifically, it is a dimeric isoenzyme of enolase, predominantly found in neurons and neuroendocrine cells. Under normal conditions, NSE participates in glycolysis within the cytoplasm of nerve cells, contributing to the physiological function of brain tissue (167). However, elevated levels of NSE can cause the degradation and rupture of the basal membrane of the BBB. The results of a clinical study by Bharosay et al. (23) showed that serum NSE concentrations were significantly associated with the degree of disability and neurological decline in AIS patients. In addition, animal studies have found that serum NSE activity IS correlated with cerebral infarct volume in rats, and the treatment of IS by regulating NSE may be related to the regulation of the TLR4/MyD88/NF-κB signaling pathway (24). Collectively, these investigations suggest that targeting NSE expression could be a promising therapeutic approach for the management of IS.

#### 3.3.5 Lipoprotein - associated phospholipase A2

Lipoprotein - associated phospholipase A2 (Lp - PLA2) is an important enzyme involved in the pro - inflammatory actions of oxidized low - density lipoproteins and is significantly associated with the progression of atherosclerosis and the occurrence of strokes (168). Research by Gorelick (169) indicated that individuals with elevated Lp - PLA2 levels had an incidence of first strokes and recurrences that was twice that of healthy controls.

## 4 Clinical applications

Inflammation is involved in all stages of IS. Accumulating evidence has demonstrated that inflammatory mediators play a crucial role in the diagnosis, prognosis, and disease management of IS. A deeper understanding of the relationship between inflammation and IS is not only beneficial for clinical application but also holds significant clinical implications.

### 4.1 Inflammatory markers and diagnosis

A research study analyzed five inflammatory markers within the first nine hours after stroke onset. These markers included MMP-9, protein S100β, von Willebrand factor, B - type neurotrophic growth factor, and MCP-1. When three or more of these markers exceeded their respective cutoff values, the result was classified as positive, yielding a sensitivity and specificity of 93% for the diagnosis of IS (158). In another study involving 889 acute stroke patients from eight original investigations, 312 patients developed post - stroke depression (PSD), while 577 did not. This research found that the serum concentrations of IL - 6 and TNF - α in the PSD group were significantly higher than those in the non - PSD group. This suggests that IL - 6 and TNF - α could potentially serve as biomarkers for PSD during the acute phase of stroke, providing a theoretical basis for the early prevention and treatment of PSD (170).

### 4.2 Inflammatory markers and disease assessment

A research investigation analyzed the variations in procalcitonin (PCT) and hs - CRP levels during the acute phase of IS, exploring the relationship between these biomarkers and long - term functional outcomes and mortality. The results showed that patients with poor functional prognoses and higher mortality at admission had significantly elevated levels of both PCT and hs - CRP. This indicates that PCT may function as an independent prognostic marker for one - year functional outcomes and mortality in patients with AIS (171).

Moreover, additional research focused on the serum levels of pro - inflammatory cytokines, such as IL - 6 and TNF - α, in IS patients, examining their potential as novel markers for predicting the functional outcomes of thrombolytic therapy. The findings revealed a correlation between the serum levels of IL - 6 and TNF - α and the degree of neurological deficits in subacute IS patients, suggesting that these biomarkers could serve as prognostic indicators for thrombolytic therapy in this context (172).

Another investigation included IS patients within 24 hours of onset and identified high - sensitivity hs-CRP, IL - 6, and TNF - α as inflammation markers at admission and 28 days after the event. This analysis concluded that elevated IL - 6 levels were associated with a poorer prognosis. Additionally, the results indicated that increased TNF - α levels in patients with lacunar infarction were related to the severity of neurological impairment at admission, suggesting that TNF - α might reflect underlying arteriolar damage (139). Youn et al. (140) investigated the relationship between hs - CRP levels and the quartile of infarction volume evaluated by diffusion - weighted imaging (DWI). They found a significant positive correlation between hs - CRP levels and DWI infarction volume. This research suggests that increased hs - CRP levels may indicate a larger CI volume and could serve as a serological marker for assessing the severity of AIS. Overall, these findings imply that elevated inflammatory markers in AIS patients may reflect the severity of the disease.

In another study, 500 AIS patients were divided into the early neurological deterioration(END) group (111 cases) and the non-END group (389 cases) according to the presence or absence of END within 7 days of onset, and serum Lp-PLA2 and MMP-9 levels and clinical baseline data were compared between the two groups. Logistic regression analysis was performed to determine the independent predictors of END, and the predictive effects of Lp-PLA2 and MMP-9 levels on END were assessed by subject work characteristics (ROC) curves. Multivariate logistic regression analysis showed that Lp-PLA2 and MMP-9 levels were independent influencing factors of END in AIS patients, and ROC curve analysis showed that the area under the curve of Lp-PLA2, MMP-9 and their combination to predict END were 0.730, 0.763 and 0.831, respectively. The inflammatory markers Lp-PLA2 and MMP-9 were both independent predictors of END in AIS patients, and the combined predictive value of the two was higher (P ≤ 0.05) (173).

### 4.3 Inflammatory markers and prediction of recurrence

Inflammatory biomarkers have demonstrated significant value in predicting the likelihood of vascular event recurrence after IS. A recent study focused on evaluating the prognostic significance of inflammatory molecular markers associated with the risk of vascular disease recurrence in IS patients not receiving anticoagulants. It was revealed that elevated levels of IL - 6, specifically those exceeding 5 pg/dl, and vascular cell adhesion molecule type 1 (VCAM - 1) exceeding 1350 ng/dl, were associated with 20 - fold and 3 - fold increases, respectively (174). Furthermore, individuals undergoing statin therapy showed a significant reduction in both vascular events and vascular - related mortality, which was associated with decreased levels of various inflammatory molecular markers, including IL - 6, MMP - 9, and cellular fibronectin (172). Additional research has shown that serum concentrations of hs - CRP are closely related to the severity of atherosclerosis and serve as a risk factor for IS recurrence at both 1 - year and 2 - year follow - ups (175).

In addition, a clinical study in China looked at the relationship between serum CXCL12 levels and stroke recurrence in AIS patients and found that elevated circulating CXCL12 levels at admission were strongly associated with IS recurrence (176). In the follow-up population, the median CXCL12 level was significantly higher in patients with relapse than in those without relapse [24.2 ng/mL (IQR 15.4-33.7) vs. 6.5 ng/mL (IQR 3.4-10.2); p < 0.05]. Z=8.258, p < 0.0001], and serum CXCL12 level ≥12.15 ng/mL was associated with an increased risk of stroke recurrence (OR 9.122, 95%CI 6.103-15.104).

A prospective study of 3401 AIS patients was conducted and serum Lp-PLA2 levels were measured. Among the 3278 patients who completed 1 year of follow-up, 188 patients had all-cause mortality, and Kaplan-Meier survival curve showed that the cumulative incidence of all-cause mortality increased in the quartile group of Lp-PLA2 content (log-rank p=0.018). These results indicate that serum Lp-PLA2 level is associated with prognosis within 1 year after AIS (177).

### 4.4 Inflammatory markers and other vascular diseases

Inflammation is a crucial mechanism in IS and also plays an essential role in atherosclerosis. Atherosclerosis is widely recognized as the primary cause of most cardiovascular diseases, such as coronary artery disease (CAD), heart failure, and stroke. The atherogenic process typically begins when cholesterol - rich low - density lipoprotein (LDL) is deposited in the endothelial lining of blood vessels, leading to endothelial activation. This activation promotes the recruitment of leukocyte adhesion molecules and chemokines, facilitating the recruitment of monocytes and T cells. Monocytes then differentiate into macrophages and upregulate the expression of pattern recognition receptors, including scavenger and toll - like receptors. The scavenger receptor is responsible for the internalization of lipoproteins, resulting in the formation of foam cells. Meanwhile, toll - like receptors transmit activation signals that trigger the release of cytokines, proteases, and vasoactive substances. In the lesions, T cells recognize local antigens, exacerbating inflammation and promoting plaque growth through the secretion of pro - inflammatory cytokines.

An enhanced inflammatory response can lead to localized proteolysis, plaque rupture, and thrombosis, ultimately resulting in ischemia and infarction (178). Research has shown that increased levels of circulating hs-CRP or IL - 6 are closely associated with the occurrence of future vascular events in both primary and secondary prevention, regardless of established risk factors (179). Moreover, additional studies have suggested that inflammatory markers are associated with the classification of IS. The median plasma levels of TNF - α, IL - 6, and IL - 1β in patients with the cardioembolic subtype of the Trial of Org 10172 in acute stroke Treatment (TOAST) were significantly higher than those in patients with lacunar IS. This indicates that inflammation may play a vital role in the development of ischemic death (180).

## 5 Therapeutic perspectives

Inflammatory markers play a crucial role in the treatment of IS and have been the focus of numerous studies. Existing research has indicated that the inhibition of cytokines, inflammatory cells, chemokines, and inflammasomes can exert beneficial effects on IS. Moreover, recent investigations have revealed that traditional Chinese medicine can be effective in treating IS by suppressing the inflammatory response. Therefore, understanding the role of inflammatory markers in the treatment of IS is of great clinical significance.

### 5.1 Inhibition of cytokines

Peripheral inflammation plays a significant role in neuroinflammation following a stroke and influences the prognosis of stroke patients. Therefore, inhibiting peripheral cytokines presents a promising therapeutic strategy. Currently, most research focuses on TNF-α inhibitors and IL-1 antagonists. The former includes drugs such as infliximab and etanercept, while the latter is represented by canakinumab. These medications are commonly used to treat inflammatory diseases, including rheumatoid arthritis and ankylosing spondylitis. Studies have demonstrated that the TNF-α inhibitor infliximab alleviates damage to the BBB following stroke in RA mice, reducing the expression of MMP-3 and MMP-9, and improving neurological function (181). Another study showed that intravenous injection of soluble TNF receptor I significantly mitigated ischemic cortical microvascular perfusion damage and cortical infarction in hypertensive rats post-stroke (182). This suggests that TNF-α inhibitors may represent a viable strategy for reducing the burden of stroke. Furthermore, a phase IIa double-blind, randomized, placebo-controlled trial involving 28 participants indicated that the initiation of sustained-release niacin tablets for 24 weeks, starting 3 to 7 days after a stroke, did not yield functional improvements (183). Additionally, administering 150 mg of canakinumab every three months for anti-inflammatory treatment targeting the innate immune pathway of IL-1β significantly reduced the recurrence rate of cardiovascular events (184). Moreover, the CANTOS trial, which also focused on cardiovascular issues, provided insights regarding its anti-inflammatory mechanism that could inform stroke prevention and treatment (185). In conclusion, while suppressing peripheral cytokine levels is crucial, current evidence supporting the use of cytokine inhibitors for treating IS remains insufficient. Further multicenter, large-scale clinical trials are necessary, and a careful evaluation of the associated risks and benefits is warranted.

### 5.2 Modulating the brain-gut axis

Numerous studies in recent years have demonstrated that cardiovascular diseases and strokes, which are associated with atherosclerosis, correlate with alterations in the intestinal microbiota and an increase in intestinal bacteria (186). Recent investigations have highlighted the pivotal role of the gut-brain axis in neuroinflammation following a stroke, suggesting that this could lead to novel therapeutic strategies for stroke prevention and treatment. Research indicates that supplementation with Lactobacillus, particularly Lactobacillus rhamnosus GMNL-89 and Lactobacillus acidophilus GMNL-133, can reduce CI volume in the MCAO mouse model and enhance neurological function, thereby exhibiting neuroprotective effects (187). Furthermore, the protective effects of Lactobacillus on IS may be linked to the inhibition of TLR-4/NF-κB signaling, the mitigation of inflammatory responses, and the suppression of neuronal apoptosis (188). Additionally, fecal microbiota transplantation (FMT) has been shown to significantly alter the composition of intestinal microbiota in IS, reduce pathogenic bacteria, increase beneficial bacteria, markedly diminish neurological impairment, eliminate brain edema, and decrease infarct size (189). Moreover, by modulating intestinal microbiota and sphingolipid metabolism, FMT can alleviate multi-organ damage caused by stroke-induced pneumonia (190). Recent studies have also indicated that vagus nerve stimulation (VNS) can rectify gastrointestinal microbiota imbalance and diminish intestinal and neural inflammation by inhibiting mast cell degranulation and reducing trypsin secretion. This regulation not only enhances the integrity of the intestinal and BBB and improves gastrointestinal function post-stroke but also mitigates systemic inflammatory responses (191). Future research should concentrate on elucidating the precise mechanisms underlying gut-brain communication after stroke and translating these insights into safe and effective therapies targeting peripheral drivers of central inflammation. Furthermore, clinical investigations have shown that probiotic supplementation may exert anti-inflammatory, antioxidant, metabolic, and gut microbiota regulatory effects in the context of cardiac remodeling (192). This suggests that the anti-inflammatory mechanisms of probiotics may represent a potential strategy for the prevention and treatment of strokes. Ultimately, the proactive prevention and treatment of infections following a stroke remains a crucial clinical strategy for alleviating adverse peripheral inflammation.

### 5.3 Regulation of glial cell polarization

Following the occurrence of IS, both microglia and astrocytes become highly activated, releasing various inflammatory factors that exacerbate brain injury. Thus, inhibiting the over - activation of microglia and astrocytes may mitigate this damage and could be a key approach in the treatment of IS.

Studies have shown that subcutaneous injection of cottonseed oil can suppress the TLR4/NF - κB signaling pathway, reduce the activation levels of neurotoxic type A1 astrocytes, and weaken the interactions between microglia and astrocytes (193). Additionally, stephanine, an alkaloid, has been found to improve the outcomes in MCAO rats by inhibiting the TLR4/NF - κB signaling pathway. This, in turn, alleviates neuronal damage and reduces microglial over - activation (194). Moreover, aleglitazar, a dual agonist targeting peroxisome proliferator - activated receptors (PPARα and PPARγ), has been demonstrated to inhibit the migration of microglia and the secretion of cytokines and adhesion molecules in MCAO rats (195).

Recently, a lipid nanoparticle approach targeting M2 microglia (termed MLNP) has been developed. This approach selectively delivers mRNA encoding phenotype - switching IL - 10 to the ischemic brain, creating a beneficial feedback loop that promotes microglial polarization toward a protective M2 phenotype (17). It also enhances the homing of MLNP loaded with mIL - 10 (mIL - 10@MLNPs) to the ischemic region. In a transient MCAO mouse model of IS, intravenous injection of mIL - 10@MLNPs induced the production of IL - 10 and enhanced the M2 polarization of microglia, thereby improving the disruption of the BBB after IS.

The activation of glial cells significantly contributes to the exacerbation of the inflammatory response. By inhibiting the over - activation of these cells and regulating glial polarization, brain damage can be mitigated, and neurons can be protected. Therefore, targeting glial cells offers a novel strategy for treating IS, and how to promote the conversion of glial cells to a favorable phenotype is worth exploring in depth next. Moreover, the related pathways and targets require further investigation.

### 5.4 Inhibition of chemokines

Chemokines play a vital role in the pathogenesis of IS, and their inhibition is beneficial for controlling the inflammatory response in IS. In a specific study, the CCR5 antagonist maraviroc (MVC, APEXBIO, UK - 427857) was administered to mice undergoing MCAO. *In vivo* experiments showed that the mice received intraperitoneal (i.p.) doses of MVC at 20 mg/kg for three consecutive days after MCAO. The administration of MVC exerted a neuroprotective effect, as evidenced by the reduction in neurological deficits and infarct volume after MCAO. Moreover, treatment with MVC significantly decreased apoptosis and inflammation levels after cerebral ischemia/reperfusion (CI/R) injury (196).

In another study, using SH - SY5Y cells and a rat MCAO model, the function and mechanism of microRNA (miR) - 532 - 5p in CI/R injury were explored. The results indicated that intraventricular injection of a miR - 532 - 5p agonist led to a reduction in CI size, lower cerebral water content, and reduced neuronal apoptosis. It also inhibited the CXCL1/CXCR2/NF - κB signaling pathway in rats with the MCAO model. These findings suggest that the overexpression of miR - 532 - 5p significantly alleviates CI/R injury both *in vitro* and *in vivo* by targeting CXCL1 (197). In summary, targeting chemokines may provide a new therapeutic strategy for IS, which merits further research.

### 5.5 Inhibition of inflammasome

During AIS, the activation of the NLRP3 inflammasome drives neuroinflammation in neurons. Research has demonstrated that early inhibition of NLRP3 can protect against ischemia/reperfusion (I/R) injury by reducing inflammation and maintaining the integrity of the BBB (198).

Robinin (5,7 - dihydroxy - 4’-methoxyflavone) is considered a promising neuroprotective compound. A study aimed to establish a cerebral ischemia - reperfusion injury model in C57BL/6 mice using a modified thread - plug technique to explore the impact of robinin on the NLRP3 inflammasome after cerebral ischemia - reperfusion injury. In this investigation, one hour after the occlusion of the middle cerebral artery, either 25 mg/kg of robinin (the robinin group) or the same volume of normal saline (0.1 mL/10 g, the MCAO group) was administered via intraperitoneal injection for reperfusion. The infarction volume and neurological function scores were assessed using 2,3,5 - triphenyltetrazolium chloride staining and the Zea - Longa scoring system. The results showed that both the neurological function scores and CI volumes were significantly lower in the robinin group compared to the MCAO group. Western blot analysis revealed that the expression levels of toll - like receptor 4, NF - κB, NLRP3, procaspase - 1, caspase - 1, pro - IL - 1β, and IL - 1β were significantly decreased in the robinin group compared with the MCAO group. These results imply that robinin may protect against cerebral ischemia - reperfusion injury, potentially by modulating the NLRP3 signaling pathway (199).

Furthermore, other research has suggested that NLRP3 inflammasomes play a critical role in mediating inflammation and pyroptosis in cerebral endothelial cells, ultimately leading to the impairment of the BBB during cerebral ischemia (CI). Studies have shown that inhibiting NLRP3 inflammasomes can reduce hypoxia - induced death of endothelial cells *in vitro* and help maintain the integrity of the BBB in rat models after a stroke (200).

### 5.6 TCM treatment of ischemic stroke

Recent research has demonstrated the effectiveness of traditional Chinese medicine in treating IS. In animal models, it can reduce both infarct volume and nerve function deficits by inhibiting the inflammatory response, and multiple paths involved. Studies have shown that ginsenoside Rg1, curcumin and notoginsenoside R1 (NG-R1) can inhibit the release of pro-inflammatory cytokines through TLR4/MyD88/NF-κb, TLR4/p38/MAPK and other pathways, reduce CI volume and improve neurological function in MCAO/R models (201–204). Quercetin (Que), baicalein and Psoralatin (PAP-1) inhibit the M1 polarization of microglia and promote the activation of M2 microglia by affecting TLR4/NF-κB, PI3K/Akt/mTOR pathway or inhibiting potassium channel Kv1.3. Finally, it shows neuroprotective properties in hypoxic-ischemic brain damage (205–207).In addition, the therapeutic effects of panax notoginseng saponins in IS have also been demonstrated in clinical trials. The meta-analysis included 46 randomized controlled trials involving 7957 AIS patients. The results showed that panax notoginseng saponins (Xuesaitong, XST) could improve long-term functional outcome by reducing the modified Rankin scale (mRS), improving activities of daily living and neurological deficits. In addition, there were no significant differences between the XST and control groups in the rates of death from any cause or adverse events (208).

## 6 Summary and outlook

Stroke presents a substantial challenge to global health, imposing a significant burden on both families and society. The inflammatory response is a crucial factor in the occurrence and development of IS. The core of acute-phase anti-inflammatory treatment lies in targeting the key pathways of neuroinflammation: by inhibiting the NLRP3 inflammasome, the maturation and release of IL-1β and IL-18 can be blocked, thereby alleviating early neuroinflammatory damage; at the same time, by antagonizing the CCL2/CCR2 or CXCL1 signaling pathways, the infiltration of neutrophils and monocytes into the brain parenchyma can be effectively inhibited, and the expansion of the inflammatory cascades can be delayed. After entering the repair phase, the reprogramming of the immune microenvironment becomes the focus: anti-inflammatory factors such as IL-4 and IL-10 can induce microglia to polarize to the M2 phenotype, promoting angiogenesis and synaptic remodeling; in addition, the activation of the CXCL12/CXCR4 axis helps recruit neural precursor cells to migrate to the ischemic area, enhancing neurogenesis and tissue repair. The entire strategy presents a logical progression from “inhibiting damage” to “promoting regeneration”, reflecting the dynamics and target specificity of neuro-immune regulation in different stages of stroke. Therefore, the development of novel anti-inflammatory or pro-reparative drugs tailored to the specific temporal phases of IS is crucial.

Furthermore, the inflammatory response within the CNS is influenced to some extent by peripheral inflammatory responses. This underscores the necessity of managing peripheral inflammation as a crucial component of IS treatment. Therapeutic strategies should extend beyond the CNS to mitigate the harmful cycle between peripheral inflammation—particularly driven by SIRS and gut dysbiosis/leakage—and neuroinflammation. Accelerating the transition from a pro-inflammatory to an anti-inflammatory phenotype, both centrally and peripherally, is of immense clinical significance. Additionally, interventions that target the gut-brain axis, such as microbiota modulation and barrier fortification, represent promising avenues for enhancing stroke outcomes. Future research must prioritize elucidating the intricate interactions within this extended neuro-immune network to identify novel, effective, and phase-specific therapeutic targets.

Research on biomarkers in IS continues to face limitations and challenges. (1)Technical Bottlenecks: Numerous potential biomarkers, such as transcriptional RNA-derived fragments (tRFs), have emerged as promising candidates for differentiating stroke subtypes. However, technical and computational challenges may compromise the reliability of tRF expression data obtained from RNA sequencing (209). Therefore, technological innovation and methodological optimization are crucial. Additionally, strict standardization and normalization of detection methods for biomarkers—including sample collection, processing, detection procedures, and data analysis—are necessary to enhance comparability and reproducibility across different studies. (2)Dynamic Changes and Time Window Challenges: The expression levels of biomarkers may fluctuate dynamically following a stroke, and their diagnostic and prognostic values may vary depending on the timing of the study (210). Utilizing appropriate biomarkers and selecting optimal blood sampling time points for diagnosing the early stages of IS are critical for facilitating more effective treatment. The development of precise and high-performance detection systems or kits will create new opportunities for rapid and accurate point-of-care testing. (3)Translational Application and Cost-Effectiveness Considerations: Many newly identified biomarkers, such as various mRNAs or autoantibodies, demonstrate favorable AUC values (e.g., the four-gene diagnostic model with AUC > 0.9) (211). However, their high costs hinder widespread adoption. 4. Heterogeneity and Individual Differences: Patients with CI exhibit significant heterogeneity in terms of etiology, lesion location, and clinical manifestations, complicating the universal applicability of a single biomarker. Addressing this issue requires close integration with clinical practice, necessitating strengthened longitudinal studies and clinical translation. Conducting large-scale, multicenter, prospective clinical cohort studies with long-term dynamic sample collection and follow-up is essential to elucidate the changes in biomarkers throughout the disease course and their association with prognosis. Furthermore, clinical research designs should be more standardized, clearly defining inclusion criteria and outcome assessment methods, while also conducting stratified studies based on disease heterogeneity to provide precise prevention and treatment evidence.

Numerous existing studies have focused on the relationship between stroke and inflammation. Future research should emphasize the following key areas: (1)The integration of inflammatory characteristics with multimodal neuroimaging. This involves combining inflammatory markers found in serum and cerebrospinal fluid with multimodal imaging techniques to analyze changes in inflammation, brain structure, and function from the acute phase to the recovery phase. Maier B.highlights the use of PET-MRI technology to identify specific inflammatory targets and visualize the spatiotemporal evolution of neural inflammation (212). Candelario-Jalil E asserts that the combination of inflammation and neuroimaging is crucial for predicting brain injury progression, assessing the extent of BBB disruption, and evaluating the potential for neural repair, thereby providing a reliable foundation for personalized anti-inflammatory treatments (213). (2)Multi-omics-driven analysis of inflammatory mechanisms. Given that stroke is a complex, multifactorial disease, regulating a single molecular factor is unlikely to sufficiently mitigate or reverse stroke pathology. In this context, integrated omics is anticipated to facilitate the identification of biologically interrelated processes in stroke. Montaner J provides an example of using multi-omics to explore therapeutic drug targets (214). Additionally, combining proteomics and metabolomics can help identify protein interaction networks and metabolic alterations in key inflammatory pathways. Montaner J. notes that transforming these findings into clinically applicable diagnostic tools or effective treatment methods necessitates a lengthy process of validation, optimization, and clinical trials. (3)AI-enabled classification of inflammation and prognosis prediction. This approach integrates clinical data (e.g., NIHSS score), inflammatory markers (e.g., CXCL12, MMP-9), and imaging features (e.g., infarction volume, white matter integrity) using deep learning techniques, such as the Transformer model, to predict the risk of stroke recurrence and functional outcomes, as proposed by Shlobin.NA (215). Furthermore, developing AI-driven treatment response models could optimize the timing and dosage of anti-inflammatory drugs based on dynamic changes in inflammatory marker levels, thereby avoiding the detrimental effects of indiscriminate anti-inflammatory therapy on recovery.
